# Trends of Tuberculosis Case Notification and Treatment Outcomes in the Sidama Zone, Southern Ethiopia: Ten-Year Retrospective Trend Analysis in Urban-Rural Settings

**DOI:** 10.1371/journal.pone.0114225

**Published:** 2014-12-02

**Authors:** Mesay Hailu Dangisso, Daniel Gemechu Datiko, Bernt Lindtjørn

**Affiliations:** 1 Center for International Health, Faculty of Medicine and Dentistry, University of Bergen, Bergen, Norway; 2 Sidama Zone Health Department, Hawassa, Ethiopia; 3 Hawassa University, Hawassa, Ethiopia; 4 Liverpool School of Tropical Medicine, Liverpool, United Kingdom; Johns Hopkins Bloomberg School of Public Health, United States of America

## Abstract

**Background:**

Ethiopia is one of the high tuberculosis (TB) burden countries. An analysis of trends and differentials in case notifications and treatment outcomes of TB may help improve our understanding of the performance of TB control services.

**Methods:**

A retrospective trend analysis of TB cases was conducted in the Sidama Zone in southern Ethiopia. We registered all TB cases diagnosed and treated during 2003–2012 from all health facilities in the Sidama Zone, and analysed trends of TB case notification rates and treatment outcomes.

**Results:**

The smear positive (PTB+) case notification rate (CNR) increased from 55 (95% CI 52.5–58.4) to 111 (95% CI 107.4–114.4) per 10^5^ people. The CNRs of PTB+ in people older than 45 years increased by fourfold, while the mortality of cases during treatment declined from 11% to 3% for smear negative (PTB-) (*X^2^*_trend_, P<0.001) and from 5% to 2% for PTB+ (*X^2^*_trend_, P<0.001). The treatment success was higher in rural areas (AOR 1.11; CI 95%: 1.03–1.2), less for PTB- (AOR 0.86; CI 95%: 0.80–0.92) and higher for extra-pulmonary TB (AOR 1.10; CI 95%: 1.02–1.19) compared to PTB+. A higher lost-to-follow up was observed in men (AOR 1.15; CI 95%: 1.06–1.24) and among PTB- cases (AOR 1.14; CI 95%: 1.03–1.25). More deaths occurred in PTB-cases (AOR 1.65; 95% CI: 1.44–1.90) and among cases older than 65 years (AOR 3.86; CI 95%: 2.94–5.10). Lastly, retreatment cases had a higher mortality than new cases (6% vs 3%).

**Conclusion:**

Over the past decade TB CNRs and treatment outcomes improved, whereas the disparities of disease burden by gender and place of residence reduced and mortality declined. Strategies should be devised to address higher risk groups for poor treatment outcomes.

## Introduction

Tuberculosis (TB) is a major public health problem in the world that affects approximately nine million people, causing deaths in approximately 1.3 million [1], with most of the cases and deaths being in sub-Saharan Africa. TB prevention and control relies on the Directly Observed Treatment, Short-term (DOTS) strategy, which focuses on case notification and successful treatment as a measure of its performance [1]. TB differentially affects different segments of the community. Some studies report a higher male to female ratio in TB case notification and lower case fatality rates in men [2]–[5]. Access to treatment, poor socioeconomic status [6], [7], health service access and use, delays in seeking care and diagnosis [8] and poor knowledge about the disease [9] all contribute to discrepancies in case notifications. A change in the occurrence of the disease by age structure, reducing the proportion of the disease by gender and urban-rural settings and trends of treatment results are important indicators to assess the effectiveness of TB control programmes [1], [10], [11].

Ethiopia is one of the high TB burden countries due to the related sickness and death [1], [12] undergoing an accelerated decentralization of DOTS, which improved case notification rate and treatment outcomes [13], [14]. Unfortunately, the case detection rate of smear-positive TB (PTB+) using passive finding which misses the less advantaged in the community [15], is below the global target of 70% [1], [13]. In such settings, an active case finding (ACF) could be useful to improve early case finding and treatment at a reasonable cost in resource-constrained settings [15]–[18]. TB case detection and treatment results vary between different regions of Ethiopia [13], which could be influenced by reports based on basic management units (BMU) regardless of their place of residence. In real life, people seek diagnosis and treatment wherever the services are accessible, available and preferred. BMU-based data aggregation includes cases from different administrative areas, and not only from the institutional catchment population. This may result in both an over- and underestimation of CNR to the true population and treatment outcomes for the defined administrative areas.

An analysis of trends and differentials in case notifications and treatment outcomes by place of residence, gender and age distribution may help improve our understanding of the performance of DOTS services and true CNR. Previous reports have focused on trends in TB case detection and treatment outcome by socio-demographic factors and treatment sites [14], [19], [20]. Nevertheless, these studies lack a further analysis based on the true address of cases, which could provide useful evidence for disease control programs for informed decision making. Therefore, the objective of this study is to assess the trends of TB case notification and treatment outcome based on the patient's address over a period of 10 years in the Sidama Zone in southern Ethiopia.

## Methods

### Study Area and Setting

This study was conducted in the Sidama Zone (the study area) in southern Ethiopia, which is one of the most densely populated areas in Ethiopia, with a population of over 3.4 million [21], [22]. Ninety-two percent of the population lives in rural areas, and agriculture is the major livelihood of the community. Administratively, the Sidama Zone is divided into 19 woredas (districts) and two towns. Additionally, there are 524 rural and 39 urban kebeles (the lowest administrative units for approximately 5,000 people or 1,000 households on average).

In recent years, there has been a substantial expansion of primary health-care services in the Zone, mostly in relation to public institutions. The public sector runs two hospitals, 102 health centers, seven non-governmental organization (NGO) clinics and 519 health posts, though private facilities were not engaged in TB care during the study period. In 2003, Ethiopia launched the health extension program, a community-based initiative to improve access to basic primary health-care services including TB, primarily focusing on households [23]. Each health post is staffed by two female health extension workers (HEWs) from the local communities, who have been trained for one year, receive salaries from the government and provide promotive, preventive and basic curative health services.

DOTS is decentralized and the DOTS providing health facilities in the study area increased from 65 in 2003 to 114 in 2012. TB and HIV collaborative activities such as HIV screening and treatment have also been carried out, and 95% of districts in the study area had at least one ART center in 2012. Health facilities (health-care services) that provide anti-retroviral treatment increased from one in 2003 to 20 in 2012 [24]. Moreover, in October 2011, the zonal health department started a community-based intervention focusing on active case finding [15] to help improve TB case finding and treatment results.

### TB Diagnosis and Treatment

We used the National TB control guidelines of Ethiopia for the diagnosis and treatment of TB cases, for case definition and treatment outcomes [25]. Health posts render health education, identify suspects, refer patients to health centers and support treatment through HEWs. Health centers conduct sputum microscopy, treatment and the referral of smear-negative and extra pulmonary cases to hospitals for further management, while hospitals provide diagnosis, treatment and in patient care services [23], [25].

### Case Definition

Smear-positive pulmonary TB (PTB+) is diagnosed with at least two positive initial sputum smears for Acid Fast Bacilli (AFB) by direct microscopy, or one positive smear for AFB by direct microscopy and culture positive or one positive smear for AFB by direct microscopy and radiographic abnormalities consistent with active TB as determined by a clinician. The laboratory keeps all positive and negative slides for external quality assurance. Quality assurance is performed regularly at the regional laboratory, and feedback is given to a reporting health facility. A previous study reported a high specificity and good agreement of sputum microscopy between peripheral and reference laboratories [26].

Smear-negative TB (PTB-) is diagnosed when the patient is presented with symptoms suggestive of TB, has at least three initial smear examinations negative for AFB, no response to antibiotics, repeat smear-negative and radiological abnormalities consistent with pulmonary TB, as well as a clinician's decision.

Extra pulmonary TB (EPTB) is diagnosed by one culture-positive specimen from an extra pulmonary site or histo-pathological evidence from a biopsy, which is based on strong clinical evidence consistent with active EPTB by a clinician's decision. However, most health facilities diagnose the disease based on a clinician's decision because there are inadequate laboratory facilities for sputum culture or histopathology.

A short course treatment regimen is given for two phases with first line fixed combination therapy [25]. The intensive phase treatment lasts for two months with Ethambutol (E), Isoniazid (H), Rifampicin (R) and Pyrazinamide (Z) followed by a continuous six-month phase with Ethambutol and Isoniazid. Since 2011, the continuation phase has lasted for four months, and uses Isoniazid and Rifampicin.

### Data Collection

The study was conducted from August 2012 to February 2013, and data were collected from all health facilities providing DOTS services from 2003 to 2012. We collected TB unit registers from all health facilities in the districts in the year of treatment.

The variables included were: address of the patient, name of the health facility, age, sex, smear result, TB category, TB classification, intensive phase drug, year of treatment, date of treatment started, last date of treatment and treatment outcome. The treatment results included: treatment completed, cured, defaulted (lost-to-follow up), died, transferred out and unknown [1], [25]. The address of patients consisted of the actual district and kebele of the patient at the time of diagnosis, and we obtained the code for each district and kebele from the Central Statistical Agency (CSA) of Ethiopia, and linked them to each case in the TB registers.

The data were entered using Microsoft Access by university graduates with experience in data management. They were trained for four days with regard to the formats, variables of interest and data collection, including practical sessions by the principal investigator (PI) and experts from the Sidama Zone Department of Health. Data were double-entered and cross-checked by the PI and experts from the Zone for the number of cases by year, facilities and districts, in addition to the correctness and consistency of the information.

We supervised the data entry for completeness and consistency on a regular basis. In the study area, TB cases are reported by the BMU, irrespective of the patient address. The BMUs are health facilities or institutions that compile and report TB cases registered for treatment to higher administrative levels. Thus, patients coming from neighboring districts or regions are reported as the cases from the reporting BMU, which forms the basis for the computation of the case notification and treatment outcomes. We linked the data from cases registered in districts where the health facility is located, though from neighboring- or other districts within the study area to their actual districts and kebeles, using CSA codes to identify the under- or over reporting of case notifications (Table 1). We also visited neighboring health facilities located outside the study area, checked for cases from the study area that were diagnosed and treated at health facilities in the neighboring areas and included them in the study if they were from the study area. Cases from neighboring region or zones diagnosed and treated in the study area were excluded from analysis, while transferred out- and transferred in cases were checked and correctly linked to their treatment outcomes. Addresses with similar names but from different locations were linked to their correct CSA codes to prevent duplication or underreporting.

**Table 1:** Differences in cases notified after linking TB cases to their correct address in the Sidama Zone, 2003–2012.

Name of the district	Cases reported by BMU	Corrected to true address	From other districts N (%)*	To other districts N (%) **	+/−	Difference N (%) ***
Aleta chuko	2,253	2,663	501 (19)	91 (4)	−	410 (18)
Aleta wondo	1,803	2,623	1,456 (56)	636 (35)	−	820 (45)
Aleta wondo town	1,999	885	85 (10)	1199 (60)	+	1114 (56)
Arbegona	650	721	108 (15)	37 (6)	−	71(11)
Aroresa	1,303	1,374	183 (13)	112 (9)	−	71 (5)
Bensa	2,933	2724	102 (4)	311 (11)	+	209 (7)
Bona	953	1,004	213 (21)	162 (17)	−	51(5)
Boricha	4,583	3,624	52 (1)	1,011 (22)	+	959 (21)
Bursa	638	978	368 (38)	28 (4)	−	340 (53)
Chire	772	954	211 (22)	29 (4)	−	182 (24)
Dale	3,446	3,707	1,024 (28)	763 (22)	−	261 (8)
Dara	1,404	1,345	141 (10)	200 (14)	+	59 (4)
Gorche	451	552	104 (19)	3 (1)	−	101 (22)
Hawassa zuriya	1,036	1,642	636 (39)	30 (3)	−	606 (58)
Hula	2,593	1,739	22 (1)	876 (34)	+	854 (33)
Loka Abaya	697	1,035	368 (36)	30 (4)	−	338 (48)
Malga	957	926	26 (3)	57 (6)	+	31 (3)
Shebedino	2,568	2,937	510 (17)	141 (5)	−	369 (14)
Wondo Genet	3,059	3,027	23 (1)	55 (2)	+	32 (1)
Wonsho	431	966	535 (55)	0 (0)	−	535 (124)
Yirgalem town	2588	1,644	26 (2)	970 (37)	+	944 (36)
Neighboring zones	216	263	216	263		47
Total	37,333	37,333	6,910 (18.5)	7,004 (18.8)		8,404 (22.6)

A total of 216 cases were cases from the study area, but treated at facilities outside the study area and included in the analysis. A total of 263 cases were excluded from analysis because they were from other districts, but treated in the study area. Therefore, 37,070 (37,333–263) cases from the study area were included in the study.

*Number of cases from other districts/number of cases corrected to true address.

**Number of cases of other districts/number of cases reported by BMU.

***Difference between number of cases reported by BMU and number of cases corrected to BMU.

To ensure the quality of the data, we checked the consistency and correctness of the entered information with the information in the TB registries. All TB unit registries were made available until the preliminary analysis, and during the exploratory analysis we looked for errors and corrected them from the registries. To help ensure the completeness and accuracy of the data, the number of cases and patient information entered in each year and health facility were checked page by page and by the year of treatment with the information in the TB registry. Personal identifiers of TB cases were coded to maintain the confidentiality of the patient information prior to analysis, and all medical records (TB unit registries) were kept in a secure place (patient records or information was anonymized and de-identified prior to analysis).

### Statistical Analysis

Descriptive statistics of trends of case notification and treatment outcomes over the past 10 years were performed for PTB+, PTB- and ETB cases. We obtained each year's population data for the study area from CSA [22], computed case notification rates using the population for different years as a denominator and notified cases as a numerator. We computed the difference between cases reported by health facilities and corresponding districts, the BMU, after linking cases to the correct address in order to acquire actual case notification rates of the study area and to find any under- or over reporting within the districts. We analyzed trends of case notification by age, gender, urban and rural residence, and looked for the evidence of age shift and trends in case notification, as well as any disparity in the trends of case notification.

Univariate and bivariate analyses were carried out to investigate the difference and association between independent and outcome variables. A logistic regression analysis was used to identify factors associated with treatment success, loss-to-follow up and mortality during treatment and control for confounding. The data were exported from Microsoft Access to SPSS version 19. We used SPSS version 19 and EpiInfo 7 for the data analysis, and P<0.05 was considered statistically significant.

### Ethical Approval

We obtained ethical clearance from the Regional Health Bureau of southern Ethiopia, in addition to a letter of support from the Sidama Zone Department of Health, to visit and obtain information from all districts and health facilities. We coded personal identifiers of cases and kept medical records in a secure place to help maintain the confidentiality of clinical information of cases prior to analysis.

## Results

A total of 37,333 cases were reported as being diagnosed and treated from 2003 to 2012 in the Sidama Zone, although 263 cases from the neighboring region or zones were excluded from analysis, thus leaving 37,070 (99.3%) cases for analysis. We identified differences between cases reported by BMU and corrected to the cases' addresses (Table 1). A total of 6,910 (18.5%) cases were reported by other BMUs, which contributed to under reporting, while 7,004 (18.8%) cases were reported from other districts by the BMUs, which contributed to over reporting. Facilities in urban areas reported a large proportion of cases from neighboring rural areas (Table 1).

Of the 37,070 cases, 16,867 (45.5%) were women and 20,193 (54.5%) were men, yielding a male to female ratio of 1.2∶1. The mean age (SD) for all cases was 29 (15) years, (male = 30 (SD 16) and females = 27 (SD 13) years). Most of the cases, a total of 22,545 (61%), were PTB+, 7,989 (22%) were PTB- and 6,464 (17%) were EPTB. A total of 31,912 (86%) cases were from rural areas, whereas 5,158 (14%) were from urban areas. Lastly, 95% (35,314 cases) were new and 5% (1641) were retreatment cases (Table 2).

**Table 2:** Characteristics of study subjects in the Sidama Zone, 2003–2012.

Characteristics	2003 N (%)	2004 N (%)	2005 N (%)	2006 N (%)	2007 N (%)	2008 N (%)	2009 N (%)	2010 N (%)	2011 (N %)	2012 N (%)	Year not mentioned (N)	Total (N)
**All cases**	2,341 (6)	2,705 (7)	2,774 (8)	2,523 (7)	3,296 (9)	4,125 (11)	3499 (9)	3,303 (9)	5,643 (15)	6,846 (19)	15(0)	37,070
**Sex**												
Male	1,322 (56)	1,489 (55)	1,537 (55)	1,417 (56)	1,798 (55)	2,208 (54)	1,974 (56)	1,880 (57)	2,981 (53)	3,576 (52)	11	20,193
Female	1,019 (44)	1,216 (45)	1,237 (45)	1,105 (44)	1,498 (45)	1,911 (46)	1,525 (44)	1,420 (43)	2,662 (47)	3,270 (48)	4	16,867
Not mentioned	0	0	0	1	0	6 (0.1)	0	3 (0.1)	0	0	0	10
**Residence**												
Urban	341 (15)	496 (18)	438 (16)	456 (18)	544 (17)	571 (14)	540 (15)	515 (16)	625 (11)	628 (9)	4	5,158
Rural	2,000 (85)	2,209 (82)	2,336 (84)	2,067 (82)	2,752 (83)	3,554 (86)	2,959 (85)	2,788 (84)	5,018 (89)	6,218 (91)	11	31,912
**Age category** *												
0–14	367 (16)	387 (14)	369 (13)	349 (14)	483 (15)	526 (13)	433 (12)	385 (12)	662 (12)	694 (10)	1	4,656
15–24	712 (31)	765 (29)	814 (30)	735 (30)	966 (30)	1,150 (28)	1,074 (31)	1,019 (31)	1,580 (28)	1,557 (23)	8	10,380
25–34	673 (29)	805 (30)	826 (30)	711 (29)	919 (28)	1,213 (30)	970 (28)	944 (29)	1,638 (29)	1,878 (28)	2	10,579
35–44	278 (12)	352 (13)	353 (13)	308 (13)	417 (13)	540 (13)	435 (13)	398 (12)	781 (14)	1,150 (17)	0	5,012
45–54	145 (6)	220 (8)	215 (8)	195 (8)	231 (7)	353 (9)	307 (9)	299 (9)	564 (10)	880 (13)	2	3,411
55–64	81 (4)	97 (4)	101 (4)	104 (4)	130 (4)	181 (4)	154 (4)	148 (4)	253 (4)	428 (6)	1	1,678
65+	59 (3)	59 (2)	70 (3)	68 (3)	96 (3)	105 (3)	92 (3)	97 (3)	162 (3)	249 (4)	0	1,057
**TB category**												
PTB+	1,358 (58)	1,586 (59)	1,574 (57)	1,434 (57)	1,734 (53)	2,502 (61)	2,286 (66)	2,156 (65)	4,056 (72)	3,851 (56)	8	22,545
PTB-	547 (23)	537 (20)	542 (20)	552 (22)	834 (25)	807 (20)	632 (18)	637 (19)	796 (14)	2,101 (31)	4	7,989
EPTB	433 (19)	577 (21)	643 (23)	526 (21)	722 (22)	799 (19)	572 (16)	504 (15)	791 (14)	894 (13)	3	6,464
Not specified	3 (0.1)	5 (0.2)	15 (0.5)	9 (0.4)	6 (0.2)	17 (0.4)	9 (0.3)	6 (0.3)	0	0	0	70
**Patient category**												
New	2,252 (96)	2,599 (97)	2,654 (97)	2,426 (96)	3,185 (97)	3,918 (96)	3,294 (94)	3,077 (93)	5,392 (96)	6,503 (95)	14	35,314
Retreatment	87 (4)	94 (4)	80 (3)	95 (4)	107 (3)	176 (4)	187 (6)	223 (7)	248 (4)	343 (5)	1	1,641
Transfer in	1 (0)	12 (0.4)	35 (1.3)	1 (0)	4 (0.1)	29 (0.7)	16 (0.5)	1 (0)	3 (0.1)	0	0	102
Others	1	0	5	1	0	2	2	2	0	0	0	13

*Age was not recorded for 297 (0.8%) cases.

### Trends in Case Notification Rates

**Figure 1:**
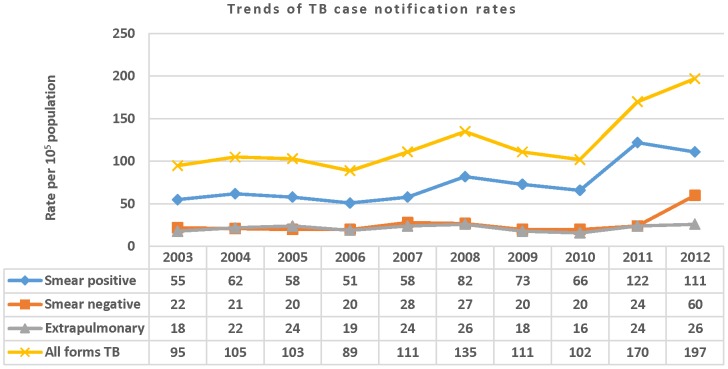
Trends of TB case notification rates per 105 people by year and by TB category in the Sidama Zone, 2003–2012.

The case notification for all forms of TB steadily increased between 2003–2012 (Figure 1). PTB+ CNR declined from 55 per 10^5^ people in 2003 to 51 per 10^5^ people in 2006 (*X^2^*_trend_, P = 0.004), while increasing from 58 (95% CI 55.8–61.3) per 10^5^ people in 2007 to 111 (95% CI 107.4–114.4) in 2012. The case notification of PTB+ in rural settings declined to 46 per 10^5^ people in 2006 (*X^2^*_trend_, P = 0.002), and increased to 110 (95% CI; 106.3–113.5) in 2012. In 2011, the CNR of PTB+ doubled for men and women compared to what it was in 2003 (Table 3). The disparity between men and women in CNR declined from 16 per 10^5^ people in 2003 to 8 per 10^5^ people in 2012, while the male to female (M: F) ratio declined from 1.4∶1 to 1.1∶1. Likewise, the CNR of PTB+ in the 45 year and above age groups rose by nearly fourfold (Table 3).

**Table 3:** Trends of smear positive TB case notification rate per 10^5^ people in the Sidama Zone, 2003–2012.

Characteristics	Year of treatment									
	2003	2004	2005	2006	2007	2008	2009	2010	2011	2012
Total PTB+ Cases	1,358	1,586	1,574	1,434	1,734	2,502	2,286	2,156	4,056	3,851
**CNRs**	55	62	58	51	58	82	73	66	122	111
**Residence**										
Urban	121	191	138	141	128	155	173	136	162	126
Rural	52	54	54	46	55	78	68	63	121	110
**Sex**										
Male	63	68	63	56	63	87	82	74	125	118
Female	47	55	54	46	54	76	64	58	118	110
**Age category**										
0–14	11	12	11	10	10	14	12	11	22	16
15–24	106	110	105	90	106	136	132	120	189	159
25–34	132	145	142	117	136	198	164	154	280	254
35–44	79	101	92	76	101	131	119	96	218	225
45–54	72	105	88	78	78	150	140	121	274	280
55–64	70	76	73	67	77	144	122	113	221	225
65+	47	49	42	51	48	70	51	77	124	135

### Trends in Treatment Outcome

Over a period of 10 years, the treatment outcome under DOTS improved among all forms, and PTB+ cases cured under DOTS decreased from 68% in 2003 to 53% in 2008 (*X^2^*_trend_, P<0.001) and increased to 89% (95% CI 87–89.1) in 2012. Moreover, cases of all forms of TB lost-to-follow up declined from 12% to 1% in 2012 (*X^2^*_trend_, P<0.001) (Table 4).

**Table 4:** Trends of treatment outcome among tuberculosis cases in the Sidama Zone, 2003–2012.

Treatment outcome	2003 N (%)	2004 N (%)	2005 N (%)	2006 N (%)	2007 N (%)	2008 N (%)	2009 N (%)	2010 N (%)	2011 N (%)	2012 N (%)	Total N (%)
**New cases**											
**PTB+**											
Completed	209 (16)	418 (26)	384 (26)	422 (31)	499 (30)	603 (26)	621 (29)	493 (25)	299 (8)	142 (4)	4,090 (19)
Cured	891 (68)	825 (54)	771 (51)	625 (46)	859 (52)	1,231 (53)	960 (45)	896 (45)	3,231 (84)	3,241 (89)	13,530 (64)
Lost-to-follow up	106 (8)	140 (9)	97 (7)	149 (11)	182 (11)	326 (14)	272 (13)	355 (18)	123 (3)	65 (1.4)	1,802 (9)
Died	69 (5.3)	52 (3.4)	40 (3)	46 (3.4)	52 (3)	50 (2)	62 (3)	42 (2)	85 (2)	72 (2)	570 (3)
Failure	4 (0.3)	1 (0.1)	1 (0.1)	0	4 (0.2)	17 (0.7)	12 (0.6)	4 (0.2)	8 (0.2)	10 (0.3)	61 (0.3)
Not evaluated*	24 (2)	83 (6)	210 (14)	122 (9)	68 (4)	112 (5)	190 (9)	197 (10)	111 (3)	125 (3.4)	1,242 (6)
**PTB-**											
Completed	367 (70)	362 (71)	376 (72)	417 (78)	659 (82)	614 (78)	445 (73)	379 (64)	683 (90)	1,850 (93)	6,152 (81)
Lost-to-follow up	78 (15)	64 (13)	54 (10)	63 (12)	83 (10)	96 (12)	92 (15)	131 (22)	27 (4)	16 (1)	704 (9.2)
Died	59 (11)	49 (10)	32 (6)	31 (6)	38 (5)	26 (3)	20 (3)	23 (4)	29 (4)	54 (3)	361 (5)
Failure	0	0	1 (0.2)	1 (0.2)	0	2 (0.3)	1 (0.2)	2 (0.3)	0	0	7 (0.1)
Not evaluated*	17 (3)	37 (7)	63 (12)	23 (4)	25 (3)	46 (6)	51 (8)	63 (11)	23 (3)	62 (3)	410 (5)
**EPTB**											
Completed	324 (76)	453 (80)	512 (82)	420 (81)	593 (83)	653 (83)	430 (77)	364 (75)	709 (92)	796 (91)	5,254 (83)
Lost-to-follow up	78 (18)	65 (12)	48 (8)	60 (12)	74 (10)	93 (12)	74 (13)	67 (14)	23 (3)	11 (1)	593 (9.4)
Died	19 (4)	13 (2)	14 (2)	16 (3)	26 (4)	14 (2)	15 (3)	20 (4)	21 (3)	14 (2)	172 (3)
Not Evaluated*	6 (1.4)	32 (6)	51 (8)	23 (4)	19 (3)	26 (3)	38 (7)	37 (8)	20 (3)	58 (7)	310 (5)
Others	0	1 (0.2)	0	0	1 (0.1)	1 (0.1)	4 (0.7)	0	0	0	7 (0.1)
**All new TB cases**											
Cured + Completed	1,791 (80)	2,059 (79)	2,043 (77)	1,888 (78)	2,611 (82)	3,104 (79)	2,461 (75)	2,135 (69)	4,922 (91)	6,029 (92)	29,043 (82)
Lost-to-follow up	262 (12)	269 (10)	199 (8)	275 (11)	340 (11)	517 (13)	440 (13)	554 (18)	173 (3)	79 (1)	3,108 (9)
Died	148 (7)	114 (4.4)	86 (3)	94 (4)	116 (4)	90 (2)	97 (3)	85 (3)	135 (3)	140 (2)	1,105 (3)
Treatment failure	4 (0.2)	2 (0.1)	2 (0.1)	1 (0)	5 (0.2)	21 (0.5)	17 (0.5)	6 (0.2)	8 (0.1)	10 (0.2)	76 (0.2)
Not evaluated*	47 (2)	155 (6)	324 (12)	168 (7)	113 (4)	186 (5)	279 (9)	297 (10)	154 (3)	245 (4)	1,968 (5.6)
Total	2,252	2,599	2,654	2,426	3,185	3,918	3,294	3,077	5,392	6,503	35,300
**Retreatment cases**											
Completed	34 (39)	38 (40)	33 (41)	49 (52)	48 (45)	65 (37)	66 (35)	78 (35)	53 (21)	146 (43)	610 (37)
Cured	30 (35)	24 (30)	21 (22)	21 (22)	28 (26)	64 (36)	56 (30)	76 (34)	163 (66)	150 (44)	647 (39)
Lost-to-follow up	7 (8)	7 (7)	5 (6)	11 (12)	15 (14)	30 (17)	30 (16)	30 (14)	8 (3)	12 (4)	155 (9)
Died	11 (13)	9 (7)	6 (8)	6 (6)	9 (8)	4 (2)	14 (8)	12 (5)	10 (4)	11 (3)	92 (6)
Failure	1 (1)	1 (1)	1 (1.3)	1 (1)	0	4 (2.3)	1 (0.5)	4 (2)	2 (0.8)	1 (0.3)	16 (1)
Not evaluated	4 (5)	4 (4)	11 (14)	7 (7)	7 (7)	9 (5)	20 (11)	23 (10)	12 (5)	23 (7)	119 (7)
Total	87	94	80	95	107	176	187	223	248	343	1,640

*Not evaluated = Transferred out cases + Cases for whom treatment outcome was unknown.

Fourteen new and 1 retreatment cases were excluded because the year of treatment was not mentioned.

One case recorded as treatment failure had no TB classification.

**Figure 2:**
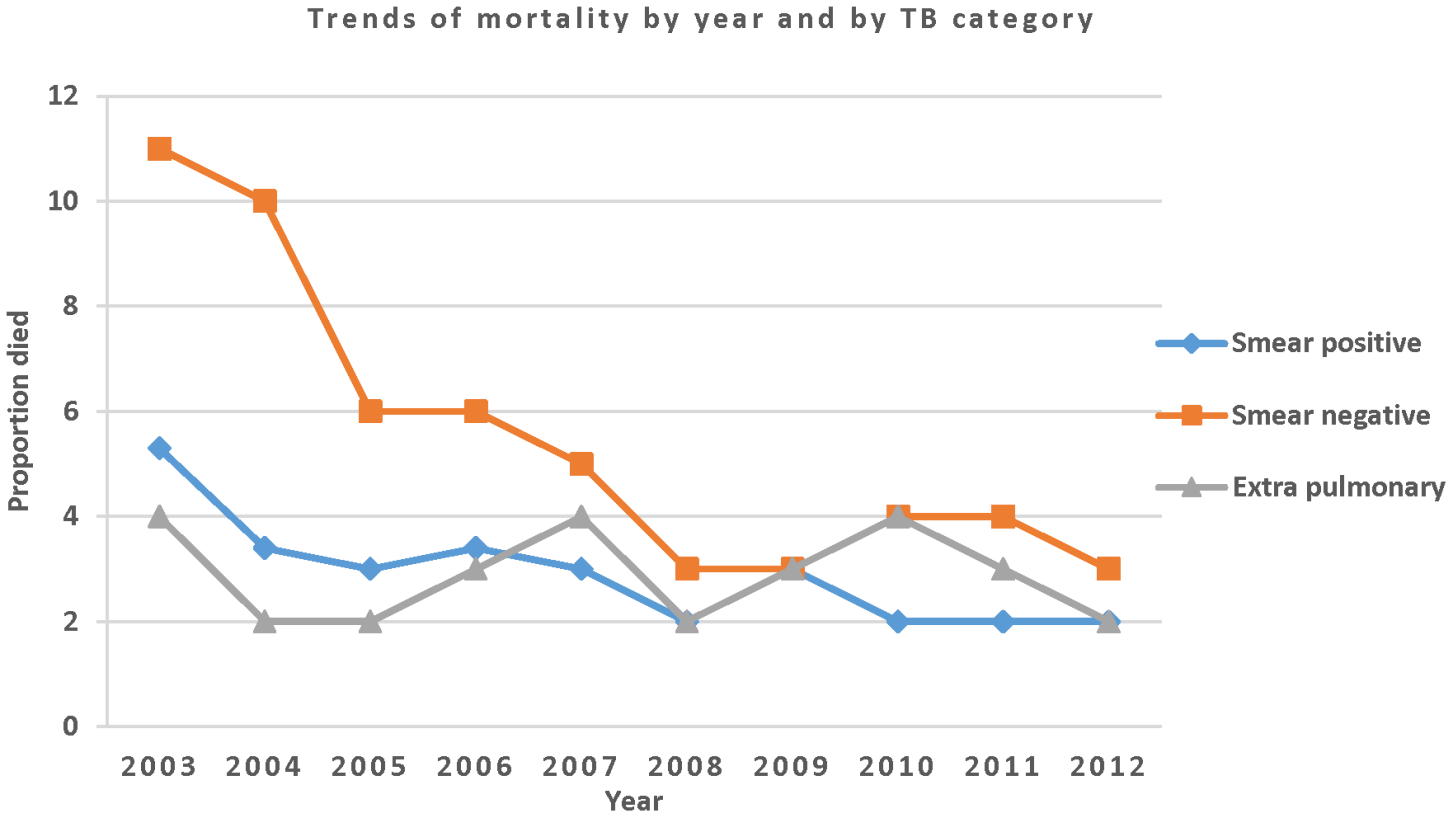
Trends of mortality among TB cases during treatment in the Sidama Zone, 2003–2012.

The proportion of cases who died during treatment declined from 11% to 3% for PTB- (*X^2^*_trend_, P<0.001), and from 5% to 2% for PTB+ (*X^2^*_trend_, P<0.001) (Figure 2 and Table 4). More deaths occurred in PTB- than PTB+ cases (AOR 1.65; 95% CI: 1.44–1.90), and similarly, cases in the age groups above 34 years and children had more deaths compared with the 15-24-year-old age group (Table 5). Additionally, patients older than 65 years were almost four times more likely to die during treatment than young adults (AOR 3.86; CI 95%: 2.94–5.10) (Table 5).

**Table 5:** Various factors associated with treatment success, loss-to-follow up and mortality among 35,314 new tuberculosis cases in the Sidama Zone, 2003-2012.

(n = 35314)	Treatment success			Lost-to-follow up			Mortality		
Characteristics	%	Adjusted OR (95% CI)	P-value	%	Adjusted OR (95% CI)	P-value	%	Adjusted OR (95% CI)	P-value
**Sex**									
Female	83.8	1.00		8.0	1.00		2.9	1.00	
Male	81	0.86 (0.81–0.91)	<0.001	9.4	1.15 (1.06–1.24)	0.001	3.4	1.07 (0.94–1.21)	0.298
**Age group**									
0–14	81.3	0.86 (0.78–0.95)	0.002	10.2	1.2 (1.09–1.40)	0.001	3	1.4 (1.09.6–1.70)	0.007
15–24	83.1	1.00		8.4	1.00		2.1	1.00	
25–34	82.6	0.92 (0.85–0.99)	0.031	8.6	1.1 (0.97–1.90)	0.163	2.8	1.37 (1.14–1.65)	0. 001
35–44	82.7	0.88 (0.8–0.97)	0.009	8.2	1.08 (0.95–1.23)	0.264	3.6	1.78 (1.46–2.21)	<0.001
45–54	82.9	0.87 (0.78–0.97)	0.010	7.8	1.06 (0.91–1.24)	0.431	4.3	2.22 (1.80–2.80)	<0.001
55–64	79.3	0.71 (0.61–0.81)	<0.001	9.8	1.3 (1.1–1.6)	0.003	6.1	3.1 (2.39–3.96)	<0.001
65+	78.6	0.72 (0.60–0.84)	<0.001	8.7	1.11 (0.87–1.4)	0.399	8	3.86 (2.94–5.10)	<0.001
**Residence**									
Urban	79.6	1.00		9.1	1.00		3.5	1.00	
Rural	82.7	1.11 (1.03–1.2)	0.010	8.8	1.1 (.97–1.21)	0.143	3.1	0.98 (.82–1.15)	0.703
**TB classification**									
PTB+	82.7	1.00		8.5	1.00		2.7	1.00	
PTB-	80.5	0.86 (0.80–0.92)	<0.001	9.2	1.14 (1.03–1.25)	0.009	4.7	1.65 (1.44–1.90)	<0.001
EPTB	82.9	1.1 (1.02–1.19)	0.016	9.4	1.03 (0.94–1.14)	0.525	2.7	0.95 (0.79–1.13)	0.532
**Year of treatment**									
2003	79.5	1.00		11.6	1.00		6.6	1.00	
2004	79.2	0.98 (.85–1.12)	0.74	10.4	0.89 (0.74–1.07)	0.20	4.4	0.66 (0.51–0.85)	0.001
2005	77.0	0.85 (0.74–0.97)	0.017	7.5	0.62 (0.51–0.75)	<0.001	3.2	0.48 (0.37–0.63)	<0.001
2006	77.8	0.91 (0.79–1.05)	0.20	11.3	0.96 (0.80–1.15)	0.656	3.9	0.50 (0.42–0.72)	<0.001
2007	82.0	1.18 (1.03–1.35)	0.02	10.7	0.88 (0.74–1.05)	0.253	3.6	0.53 (0.41–0.68)	<0.001
2008	79.2	0.99 (0.87–1.13)	0.912	13.2	1.14 (0.97–1.34)	0.112	2.3	0.33 (0.26–0.44)	<0.001
2009	74.7	0.75 (0.66–.85)	<0.001	13.4	1.19 (1.01–1.40)	0.042	2.9	0.44 (0.33–0.57)	<0.001
2010	69.4	0.58 (0.51–0.66)	<0.001	18.0	1.68 (1.43–1.97)	<0.001	2.8	0.40 (0.30–0.53)	<0.001
2011	91.3	2.6 (2.3–3.05)	<0.001	3.2	0.26 (0.21–0.31)	<0.001	2.5	0.36 (0.29–0.46)	<0.001
2012	92.7	3.3 (2.9–3.8)	<0.001	1.2	0.09 (0.07–0.12)	<0.001	2.2	0.26 (0.21–0.34)	<0.001

Cases from rural areas had a higher treatment success (AOR 1.11; CI 95%: 1.03–1.2) than cases from urban areas, whereas the treatment success was less for PTB- cases (AOR 0.86; CI 95%: 0.80–0.92) and more for EPTB cases (AOR 1.10; CI 95%: 1.02–1.19) compared with PTB+ cases (Table 5). Significant differences were also observed in treatment success, loss-to-follow up and mortality between years of treatment (Table 5), and both men (AOR 1.15; CI 95%: 1.06–1.24) and smear-negative cases also had higher loss-to-follow up rates (AOR 1.14; CI 95%: 1.03–1.25).

## Discussion

We found an increased case notification rate, improved treatment success and a decline in poor treatment outcomes over the past 10 years. However, we noted varying CNRs and treatment outcomes during the study period, and we also found less urban-rural and gender discrepancies in case notification rates.

Globally, gender inequalities have been reported in TB case notification [2], with the male to female ratios ranging from 1.5∶1 -2.2∶1[5], [27], but in contrast, lower male to female ratios were reported from Asia [1], [28]. The male to female ratios in our data were lower than reports from other studies. The decline in male to female ratio and a narrowing gender disparity gap in our data could be attributed to an improved access to TB control services [15] and active case finding interventions [15], [16], while gender differences in case notification could be due to access to health services [29], biological-, socio-economic- and cultural factors [27], [30]–[32], as well as poor knowledge and diagnosis delays [9]. Nonetheless, because active case finding have exhibited similar trends for both men and women, we believe that access to health services was the primary reason for improving gender differences.

Furthermore, we found a higher CNR of PTB+ in urban- than in rural settings, which could be explained by adverse conditions such as poor socioeconomic status [33], [34], overcrowding [7] and a high prevalence of HIV/AIDS [35]. Previous studies from southern Ethiopia reported higher TB and HIV co-infection rates (25–30%) in cases from urban settings [36], [37], and the demographic and health survey of Ethiopia also reported a higher prevalence of HIV in urban- than in rural settings [38].

We found a steady increase in CNR among younger groups and a more than twofold increase in older groups. However, no age groups showed a decreasing trend in CNR over the past decade. In developed countries, the age shift occurred over a period of years, with TB being common in older age groups due to an aging population and the changing epidemiology of the disease [39]–[41]. In developing and high burden countries, the disease is common among younger age groups [1], [41]. The increasing trend in CNR in the 65 year olds in our study was consistent with a study from South Korea, though unlike our finding, the proportion of notified TB cases in South Korea for those under the age of 65 decreased [39]. This difference could be due to population distribution because the proportion of notified cases over 65 years in Korea was higher than notified cases over 65 years in our study, which could also be because of active case finding interventions in the study area [15], [16]. The increase in CNR among the older age group in our study indicates TB among the old has been under-diagnosed, while the observed increase in CNRs during the study period could be attributed to improved access to- and utilization of TB control services [15], [16]. Hence, the DOTS services and community-based interventions in the study area have an improved access to- and use of TB services, as well as a reduced under-diagnosis of TB among older age groups. Nevertheless, increasing aging is related to increased risk due to a reduced immunity and co-morbidities such as diabetes, malignancies and other factors that increase cases [39], [41]. The increased CNR among older age groups in our study should be interpreted with caution because studies on age shift in TB occurrence report an age shift after a long study period usually lasting for decades.

Treatment success was poor in 2008–2010, although a notable improvement was observed in 2011–2012. This improvement could be due to an improved access to TB control services, particularly community-based interventions [15], early diagnosis and treatment of the cases, reduction in lost-to-follow up and a decline in the mortality of patients while on treatment. Similarly, a remarkable decline in lost-to-follow up of cases in 2011–2012 could be explained by community-based interventions, which improved access to- and the use of TB control services. Other possible reasons for a notable reduction in lost-to-follow up could be the drop in the proportion of non-evaluated cases compared to preceding years, the expansion of TB treatment centers and a regimen change of the continuation phase to RH, which lasts four months.

In our study, more men were lost-to-follow up than women, with this finding consistent with other studies [42], [43]. PTB- cases were associated with a higher loss-to- follow up, which is consistent with a previous study from southern Ethiopia [44]. Conversely, a study from Nigeria reported a higher loss-to- follow up among PTB+ cases [42]; this difference could be due to a longer study period, a larger cohort of TB cases and wider geographic settings in our study.

The proportion of cases who died on treatment declined during the study period; however, unlike other studies, there were no significant differences in mortality by gender and urban-rural settings [45]. This could be explained by improved access to, and utilization of TB control services and an improved awareness of the disease. The proportion of cases who died on treatment among the age group of 65 years and above in our data was fourfold compared to younger adults. This finding was in agreement with the study from southern Ethiopia, which reported a higher mortality rate among the elderly [44]. This could be due to an increased risk of co-morbidities and delay in diagnosis and treatment among older age groups, which could increase adverse treatment outcomes [41].

The higher proportion of deaths in PTB- cases than PTB+ and EPTB cases in our data might be due to diagnosis and treatment delays, as well as HIV infection among PTB- cases. Studies reported more PTB- cases among HIV-infected individuals [36], [46], while previous studies from southern Ethiopia also reported a high TB-HIV co-infection, with the rate of HIV infection among PTB- cases being 18–26% [36], [37]. The declining trend in mortality during treatment in our study may be explained in part by ART services because ART reduces deaths in TB cases infected with HIV [47]. In recent years, there has been an increase in both ART centers and the number of patients starting ART in the study area [24].The number of ART centers in the study area increased from one to 20 during the study period. More deaths were also observed among retreatment cases, which is consistent with other reports [48], [49]. Retreatment cases could be drug resistant TB, repeated infection, a recurrence due to a reduced immunity and other co-morbidities that could increase the risk of death.

In our study, we found variations in cases notified by BMU in relation to place of residence, which might mask the real epidemiology of TB in the community. We noted both over- and underreporting of cases within districts in study area; the over reporting was mainly observed from urban health facilities, with the underreporting occurring at neighboring rural areas. This could be attributed to poor access to health facilities that provide TB services for rural areas in their catchment since people use the closest health facilities regardless of their actual address. If health facilities fail to correctly record and report the actual address of patients, and include or miss cases in the numerator of case notification, it results in an under- or over reporting of cases. On the other hand, people may bypass the closer health facility and use health services further away because of patient preference due to poor service delivery in the nearby health facilities or the distribution of health facilities that did not consider the population distribution and required coverage. This finding is important for the TB control program and district authorities because the over- or underreporting of cases may conceal a true burden of the disease, which may lead to flawed conclusions for targeting interventions and resource allocation. Data aggregation based on the correct address of cases could help solve the problem of over- or under-reporting. Thus, improving the recording of the actual patient address and access to TB control services might solve such discrepancies in actual case notification.

One limitation of our study is that patients with no address could be missed; however, the percentage of patients with a missed address and information was too small to affect the results. PTB- and EPTB could also be over- or underestimated because of poor access to standard diagnosis facilities such as culture and histopathology. Information about HIV status and treatment was not available in the registers; consequently, we could not compare the treatment outcomes based on HIV status and treatment. In addition, our study was not a population-based survey, so cases occurring in the community may remain undiagnosed, and a few may remain unregistered. The strengths of our study were that the study was carried out on a large cohort of TB cases, covering a large geographic area and both urban and rural settings. Lastly, this is the first study from Ethiopia that uses patients' true home addresses to assess the results of a tuberculosis control program, as previous studies have been based on reports from BMU that did not consider the actual address of cases.

## Conclusions

We have reported that over the past 10 years, TB case notification rates increased, poor treatment outcomes were reduced, while disparities of the disease burden by gender and place of residence also declined. Understanding the epidemiology of TB in such settings is important for resource planning, monitoring and understanding the burden of the disease in the community. Strategies should therefore be devised to address higher risk groups for poor treatment outcomes.
