# Correction: Trends of Tuberculosis Case Notification and Treatment Outcomes in the Sidama Zone, Southern Ethiopia: Ten-Year Retrospective Trend Analysis in Urban-Rural Settings

**DOI:** 10.1371/journal.pone.0125135

**Published:** 2015-04-13

**Authors:** The PLOS ONE Staff

There is an error in the legend for Fig 1. The publisher apologizes for the error. Please see the complete, corrected Fig 1 here.

**Fig 1:**
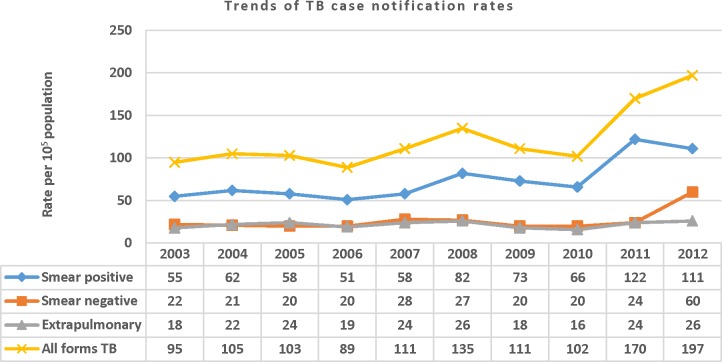
Trends of TB case notification rates per 10^5^ people by year and by TB category in the Sidama Zone, 2003–2012.
